# An unusual case of rapidly progressive contractures: Case report and brief review

**DOI:** 10.4103/0972-2327.41882

**Published:** 2008

**Authors:** R. Subasree, Samhita Panda, Pramod Kumar Pal, S. Ravishankar

**Affiliations:** Department of Neurology, NIMHANS, Bangalore, India; 1 Department of Neuroimaging and Interventional Radiology, NIMHANS, Bangalore, India

**Keywords:** Myositis ossificans progressiva, muscle, contractures

## Abstract

An 8-year-old boy, diagnosed as cervical dystonia, was referred to our tertiary center. After a trivial trauma he had developed painful lumps in the axial region, which was followed by restricted movements of neck, shoulder, and abdominal muscles over 4 months. He had kyphoscoliosis, torticollis, rigid abdomen, and multiple muscle contractures. He also had short great toes. A detailed skeletal survey showed calcification in the soft tissues surrounding the shoulder anterior chest wall, thorax, and paraspinal muscles; there was also beaking of vertebrae, which was confirmed by CT thorax. This report showcases the diagnostic challenge posed by myositis ossificans progressiva, which can rarely cause rapidly progressing muscle contractures. A brief review of literature is also presented.

## Introduction

Muscle contractures are seen in various neuromuscular and non-neurological conditions, such as Emery-Dreifuss dystrophy, calcinosis universalis, Weber-Christian disease, Bethlem myopathy, arthrogryposis, dystrophinopathies, and rigid spine syndrome. Myositis ossificans is a rare, progressive, crippling disorder, with an incidence of less than 1 in 1,000,000 population. Guy Patin first described this entity in 1692. It is synonymous with fibrodysplasia ossificans, Munchmeyer's disease, Munchmeyer's syndrome, stiff-man syndrome, and progressive ossifying myositis.[1] Around 700 cases have been reported in world literature to date. We report a young boy who presented with very rapid progression of disabling muscle contractures, which was initially misdiagnosed as cervical dystonia and later diagnosed clinico-radiologically as myositis ossificans progressiva (MOP).

## Case Report

An 8-year-old boy born to third-degree consanguineous parents presented with restriction of movements and posturing of neck and shoulder for 4 months. The child was referred to our center as a case of cervical dystonia. A detailed evaluation revealed prior history of multiple painful erythematous lumps in the neck, submandibular region, scapula, and trunk after a trivial trauma. All these lumps had subsided spontaneously after 4 months. He gradually developed restricted mobility of the left shoulder, followed by neck and right shoulder, over the next 4 months. There was no history of fever, bleeding tendencies, hematuria, seizures, deafness, mental retardation, joint swelling, rash, abdominal colic, fractures, thyroid swelling, or any drug intake. Birth and developmental milestones were normal. Family history was noncontributory.

**Figure 1:**
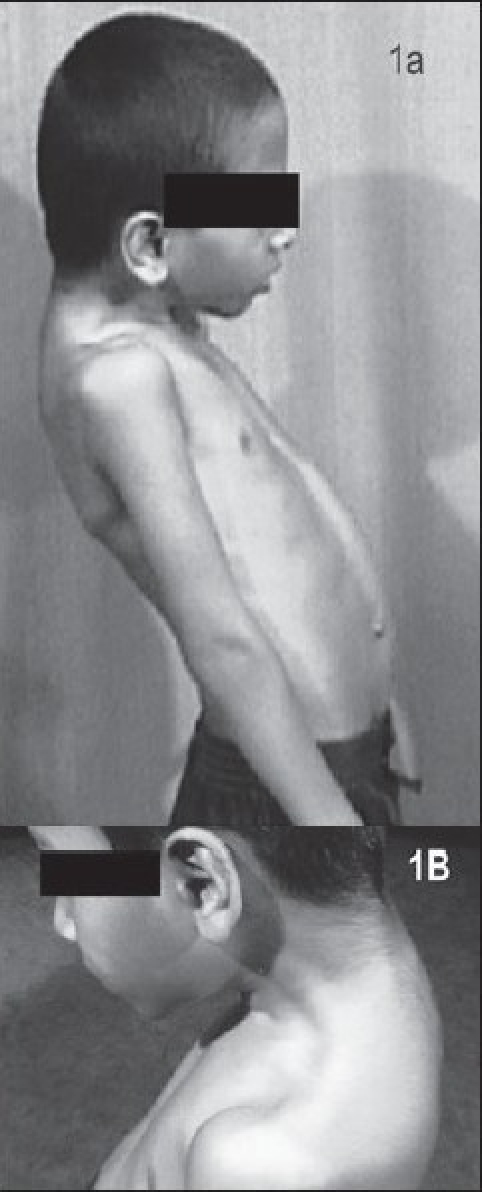
(A) Profile of the patient showing kyphosis, anterocollis, and exaggerated lumbar lordosis. (B) Flexion deformity of the neck. Prominent contractures of the trapezius and sternocleidomastoid are seen

Examination revealed normal intelligence. There was dorsal kyphoscoliosis [Figure 1A], torticollis, rigid abdomen, and obliteration of lumbar lordosis. There were muscle contractures involving the sternocleidomastoids, latissimus dorsi, pectoralis major, and the cervical muscles, with restricted abduction and internal rotation of both shoulders [Figure 1B]. He also had short great toes. The rest of the nervous system was normal. There was no respiratory or sphincteric involvement.

**Figure 2:**
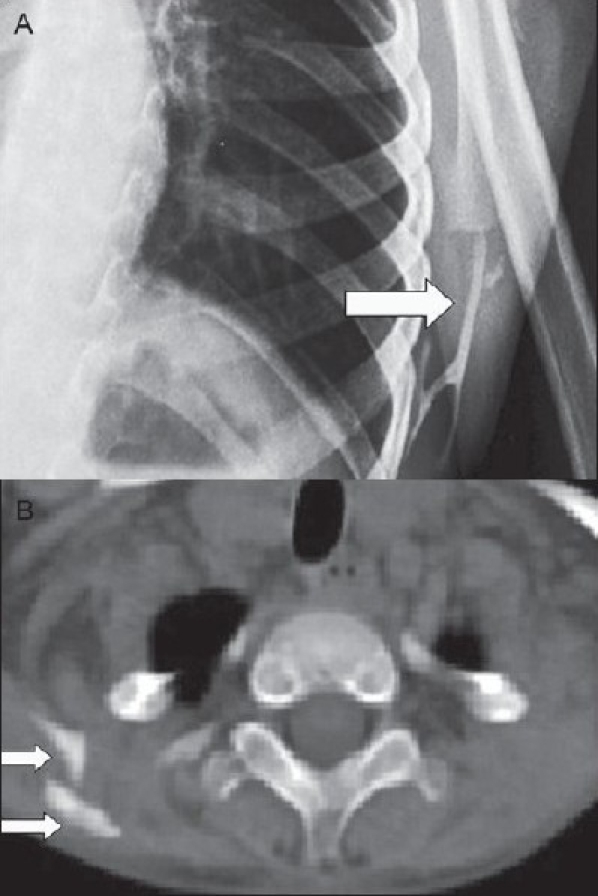
(A) X-ray chest showing calcific strands (arrow) around the inferior angle of the scapula. (B) CT thorax showing multiple plaque-like calcifications (arrows) in the left thoracic wall and paraspinal muscles

Investigations showed normal calcium (10.1 mg%), phosphorus (5.9 mg%), alkaline phosphatase (227 mg%), and erythrocyte sedimentation rate (4 mm in 1 h; Westergren's method). The spirometry and electrocardiogram were normal. A diagnosis of a dystrophinopathy such as Emery-Dreifuss was initially considered. However, the creatine kinase level was normal (95 U/l), which is unusual for a dystrophinopathy. The child was further evaluated with x-ray cervical spine; this showed calcific strands around both shoulder joints and in the paraspinal regions. A detailed skeletal survey of the body revealed calcification in the soft tissues surrounding the shoulder, in the anterior chest wall [Figure 2A], the thorax, and the paraspinal muscles; also there was beaking of vertebrae. CT thorax showed multiple plaque-like calcifications involving the pectoralis major and paraspinalis [Figure 2B]. Considering both the clinical and the radiological features, sporadic MOP was diagnosed. The child was treated with graded physiotherapy. Bisphosphonates were considered but the patient could not afford the cost involved. As there was no acute flare-up, steroids were not given. The patient has been subsequently lost to follow-up.

## Discussion

The case presented here highlights the high probability of misdiagnosis in patients of MOP. Presentation with multiple contractures may easily simulate axial and limb posturing, leading to the diagnosis of dystonia, as occurred in the present case. However, careful history taking and physical examination can clarify the issue. The condition can also be confused with muscular dystrophies such as Emery-Dreifuss dystrophy as well as with conditions like calcinosis universalis, Weber-Christian disease, Bethlem myopathy, arthrogryposis, cancer, and rigid spine syndrome. Knowledge of the clinical profile of MOP is necessary to differentiate it from these other diseases.

Diagnostic errors have been documented in up to 87% of MOP cases worldwide.[2] This high rate of diagnostic errors does not seem to be influenced by ethnicity, geographic background of the patient, or the expertise of the physicians who attended to the patient. Diagnostic errors can alter the natural history of an illness such as MOP and cause iatrogenic harm. Cancer is the most common incorrect diagnosis (32%). Interestingly, in this series, the mean period from onset of symptoms to correct diagnosis was 4.1 years, and the median number of physicians consulted before the correct diagnosis was arrived at was 6. Two-thirds of the patients underwent biopsies and half developed permanent loss of mobility due to posttraumatic ossification. In another series of patients referred for rehabilitation with a diagnosis of spinal cord injury, 12% were found to have MOP.[3]

Our case presented in the first decade of life with trivial trauma as the inciting factor. Though the child had inflammatory swellings after trauma, exceptionally, no fever was noted. The rate of development and progression of contractures over 4 months is also very rapid compared to the slow progression commonly reported in literature. As a result of the relatively rapid progression of the disease, he developed more widespread involvement of axial and limb musculature in the first decade of life itself. This highlights the phenotypic variability in the mode of presentation of MOP and its progression.

Myositis ossificans is subclassified into myositis ossificans circumscripta (MOC) and MOP. MOP is a mesodermal disorder characterized by an aberrant reparative process, which causes benign heterotopic calcification and ossification of striated muscles, ligaments, soft tissues, tendons, fascia, aponeurosis, and joint capsules; this leads to contractures and progressive skeletal deformities.[4] The approximate age of onset of ectopic bone formation is 2–6 years, ranging from birth to 25 years.[5] The case discussed here also presented in the first decade. The condition has a male preponderance.

Initial symptoms include painful lumps, mostly in the neck, dorsal trunk, and proximal extremities; there is also stiffness in the adjoining joints, with erythema and mild fever.[4] The lumps decrease in size over a few weeks but joint mobility remains restricted, as inflammatory tissue is replaced first by cartilage and then bone. The commonest presenting symptom is torticollis, with painful and warm erythematous masses in the sternocleidomastoid muscles. Locked jaw due to involvement of the masseter and pterygoids may be seen.[6,7] The sternocleidomastoid, paraspinal, masticatory, shoulder, and pelvic girdle muscles are commonly involved in the disease. Those usually spared are the abdominal muscles, heart, diaphragm, facial and extraocular muscles, and the tongue and laryngeal muscles. The sphincteric muscles and those in the gastrointestinal tract, as well as skeletal muscle in parts distal to the elbow and knee, are rarely involved. Our patient had predominant involvement of the paraspinals, sternocleidomastoid, latissimus dorsi, pectoralis major, and cervical and abdominal wall muscles. Ossification progresses from proximal to distal and cranial to caudal regions. Factors exacerbating ossification at new sites include blunt trauma, stab wounds, fracture-dislocation, and ischemia; even very minor trauma, such as venipuncture, biopsy of lumps, IM injections, dental treatments, or excision of masses can precipitate calcification, as observed in our patient.[1] Although MOC can also follow mild trauma, the lesions are usually single, spares other parts of the body, and do not recur following excision.

Associated skeletal features of great diagnostic significance include short hallux with synostosis, hallux valgus (75–90%), and short thumbs.[4] Kyphoscoliosis, with restricted shoulder and pelvic girdle movements and restrictive pulmonary disease, can occur. Conductive deafness due to ossicular fusion (25%), mental retardation, alopecia, and cardiac conduction defects are other associations.[4]

Radiological investigations help in clinching the diagnosis of MOP. The findings on skeletal survey of the extremities include clinodactyly, microdactyly of big toes (90%) and thumbs (50%), phalangeal shortening (usually the middle phalanx of the fifth digit), shortened first metatarsal and hallux valgus (75%), shortened metatarsals and metacarpals, shallow acetabulum and short widened femoral neck, and thickened medial cortex of tibia.[8] Spinal x-rays show progressive fusion of the posterior arches of the cervical spine; narrowed anteroposterior diameter of cervical and lumbar vertebral bodies, with or without bony ankylosis; small vertebral bodies; and pedicle thickening. Different bone maturation sequences are noted over the involved muscles and there is increased incidence of enchondromas.[8] Computed tomography demonstrates fascial plane edema and swelling, even before ossification has occurred. In the present case, calcification was seen in the affected areas as early as 8 months after onset of symptoms. On magnetic resonance imaging, the findings depend on the age of the lesion. In immature lesions, T2-weighted spin-echo images are associated with a homogeneous soft tissue mass with increased signal intensity. Surrounding edema may be seen in lesions less than a few months old. In T1-weighted images, only a mass effect may be noted, with displacement of fascial planes. Mature lesions appear as inhomogeneous masses with fat-like signal intensity on both T1-weighted and T2-weighted images.[8] Bone scintigraphy reveals enhanced uptake in immature lesions. Once maturation occurs, the uptake becomes comparable with that of normal bones. Ultrasonography shows echogenic and shadowing masses.

A wide range of conditions has to be differentiated from MOP. These include Weber-Christian disease (relapsing, nodular nonsuppurative panniculitis), Klippel-Feil syndrome, rheumatoid arthritis, dermatomyositis of childhood, systemic sclerosis, calcinosis interstitialis ossificans, Albright hereditary osteodystrophy, pseudohypoparathyroidism, hypervitaminosis D, traumatic myositis ossificans, multiple exostosis, and extraskeletal osteosarcoma. Radiologically, MOP is different from calcinosis interstitialis ossificans and multiple exostosis. In osteosarcoma, calcification starts from the center in association with the diaphysis. Normal calcium and phosphorus levels rule out metastatic calcification.

The genetics of MOP is largely uncertain because the disorder is likely to be lethal before the affected individual has offspring. MOP has a predominantly autosomal dominant inheritance, with complete penetrance but variable expression.[9] However, almost 95% of cases are sporadic due to a high level of spontaneous mutations. Increased paternal age has been noted in a large series of sporadic cases, suggesting new mutations in the sperm.[10] The pathogenesis of MOP is unclear as there are no biochemical markers. Linkage to chromosome 4q27-31 has been implicated.[11] This leads to excessive stimulation of bone morphogenic protein IV and its mRNA, which is involved in bone formation. The presence of type I collagen in bone, fascia, tendon, and ligament leads to its involvement and alteration by fibroblast modulating cell surface protein. Another hypothesis may be a lack of circulating inhibitor or a primary defect in collagen.[12] This can explain the deposition of calcium salts in masses of collagen as irregular lamellar and woven bone.

To date, there is no effective therapy that can impede progression in MOP. Corticosteroids are useful in the acute stages. Bisphosphonates, oral or intravenous, are a therapeutic alternative to reduce ectopic calcifications by preventing formation and causing dissolution of calcium apatite crystals.[13] Other options include ethane-1-hydroxy-1-diphosphate and isotretinoin. Some newer and investigational drugs include antiangiogenic agents such as squalamine, thalidomide, fluoroquinolones, prostaglandins, COX-2 inhibitors, BMP4 antagonists, and noggin and gremlin gene therapy. Recently, a recurrent mutation has been reported in the glycine-serine activation domain of activin receptor 1A, a bone morphogenetic protein type I receptor, in all cases of sporadic and familial MOP. This discovery uncovers a potentially critical target for future therapeutic intervention in patients with MOP.[14] Apart from these drugs, immobilization for 2–4 weeks, followed by gradual increase in exercises is usually recommended. Surgical release of contractures is recommended only in sporadic MOP after 6–24 months and only if joint movement is severely impeding movement or there is nerve impingement. There is a 50% theoretical risk of transmitting the MOP gene defect, irrespective of gender. However, the risk of a second affected child in the family is low unless one of the parents also possess short great toes.

The natural history of patients with MOP demonstrates that stiffness of the spine and shoulders develop by 10 years and pelvic movements are restricted by 20 years. The patients become confined to the bed by 30 years. Death can occur prematurely by the second to fourth decade, usually due to restrictive lung disease and respiratory failure.[15] Temporomandibular joint disease, leading to starvation deaths, developed in 70%. Rarely, patients have survived till the seventh decade.

## Conclusion

This case demonstrates a rare case of MOP with extremely rapid involvement of the axial musculoskeletal system. In addition, it highlights the high probability of diagnostic errors in this condition. In routine neurological practice the diagnosis of myositis ossificans is often missed. A high degree of suspicion and alertness is required to diagnose this rare disorder. Needle EMG and biopsy, the diagnostic tools for neuromuscular disorders with muscle contractures, is absolutely contraindicated in such cases.
